# A biosegmentation benchmark for evaluation of bioimage analysis methods

**DOI:** 10.1186/1471-2105-10-368

**Published:** 2009-11-01

**Authors:** Elisa Drelie Gelasca, Boguslaw Obara, Dmitry Fedorov, Kristian Kvilekval, BS Manjunath

**Affiliations:** 1 Center for Bio-Image Informatics, Electrical and Computer Engineering Department, University of California Santa Barbara (UCSB), CA 93106, USA

## Abstract

**Background:**

We present a biosegmentation benchmark that includes infrastructure, datasets with associated *ground truth*, and validation methods for biological image analysis. The primary motivation for creating this resource comes from the fact that it is very difficult, if not impossible, for an end-user to choose from a wide range of segmentation methods available in the literature for a particular bioimaging problem. No single algorithm is likely to be equally effective on diverse set of images and each method has its own strengths and limitations. We hope that our benchmark resource would be of considerable help to both the bioimaging researchers looking for novel image processing methods and image processing researchers exploring application of their methods to biology.

**Results:**

Our benchmark consists of different classes of images and *ground truth *data, ranging in scale from subcellular, cellular to tissue level, each of which pose their own set of challenges to image analysis. The associated *ground truth *data can be used to evaluate the effectiveness of different methods, to improve methods and to compare results. Standard evaluation methods and some analysis tools are integrated into a database framework that is available online at .

**Conclusion:**

This online benchmark will facilitate integration and comparison of image analysis methods for bioimages. While the primary focus is on biological images, we believe that the dataset and infrastructure will be of interest to researchers and developers working with biological image analysis, image segmentation and object tracking in general.

## Background

Quantitative measures derived from microscopy images are basic to enhancing our understanding of biological processes. With the rapid growth in emerging imaging technologies and high throughput bioimaging, robust image processing methods are critically needed in such quantitative analysis. While there is a large amount of literature concerning basic image processing methods, there exists currently no proper guidance for an end-user to choose a small subset of methods that are likely to be effective in a given application scenario. This is particularly true for segmentation and tracking, where literally hundreds of new methods are proposed each year. In most of these cases experimental results are provided on a very limited set of data, often coming from different domains, making it more difficult to judge their usability. The lack of well defined data sets that allow a fair comparison of different basic methods is a major bottleneck for progress in bioimage analysis. This is the main motivation in building the biosegmentation benchmark infrastructure and dataset collection for biological image analysis applications. In particular, we have collected datasets of different modalities and scales and carefully generated manual *ground truth *that could be of significant help not only to researchers in biological image analysis but also to the image processing community in general. By having a standardized set of data with associated *ground truth*, we believe that rapid progress can be made not only in identifying the appropriate methods for a particular task but also in facilitating the development of new and more robust methods.

In this paper we focus specifically on a benchmark dataset for image segmentation and tracking. Typical challenges in developing robust bioimage analysis methods include low signal to noise ratio, complex changes in object morphology and the diversity of imaging techniques (such as confocal, bright-field, electron microscopy, phase contrast imaging). Given this diversity in imaging methods and bioimage samples, it is now well recognized that there is a clear need for validating new image analysis methods, see for example [1,2].

Benchmarks can be invaluable tools for both image processing specialists and scientists. The developers of the algorithms can use such benchmarks to evaluate the performance, reliability and accuracy of newly developed methods. The benchmark provides them with a well established problem set. Further, the workload involved in validation can be reduced significantly by providing access to other analysis and evaluation methods [1].

There have been several successful benchmarking efforts in image analysis and computer vision, such as the face recognition dataset [3], the Berkeley (University of California Berkeley) segmentation dataset for natural images [4] and the object Caltech (California Institute of Technology) 101 dataset [5]. In medicine, databases with macrobiological structures such as mammogram and Magnetic Resonance images [6], and clinical data [7] have also been developed. In biology, there have been some efforts in creating microbiological image databases such as the Cell Centered Database [8] and the Mouse Retina Database [9]. The Protein Classification Benchmark Collection [10] was created in order to collect a standard datasets on which the performance of machine learning methods can be compared. Finally, the Broad Bioimage Benchmark Collection [11] consists of microscopy image sets with associated *ground truth*, such as cell counts, foreground/background and object outlines.

In addition to the above datasets, there have been few organized competitions in computer vision. These include the Face Recognition Grand Challenge (FRGC) [12], Face Recognition Vendor Test (FRVT) 2006 [13], and the Iris Challenge Evaluation 2006 [14]. Data and evaluation results of Iris Recognition competition are available in [15] and Benchmarking Change Analysis Algorithms in Lung CT in [16]. Results from FRGC and FRVT 2006 challenges documented two orders of magnitude improvement in the performance of face recognition under full-frontal, controlled conditions over the last 14 years. Similarly, researchers have reported a significant improvement in object recognition performance over the Caltech 101 and Caltech 256 datasets over the last few years. This further supports our earlier observation that good benchmark datasets with ground truth information can act as catalysts in the development of robust image analysis methods.

A preliminary version of this dataset was presented in a conference publication at the International Conference Image Processing'08 [17]. This work expands on [17] by providing detailed descriptions on segmentation algorithms and performance metrics. In addition a new 3D image dataset is included. Also, we describe a web-accessible infrastructure that we have developed recently for testing the algorithms. A flexible metadata model (see Section Availability and Requirements and Appendix) is described that is used to exchange data and results for performance evaluation. This infrastructure lowers the burden of choosing datasets for testing algorithms, re-implementing analysis methods and developing evaluation metrics for comparison.

**Figure 1:**
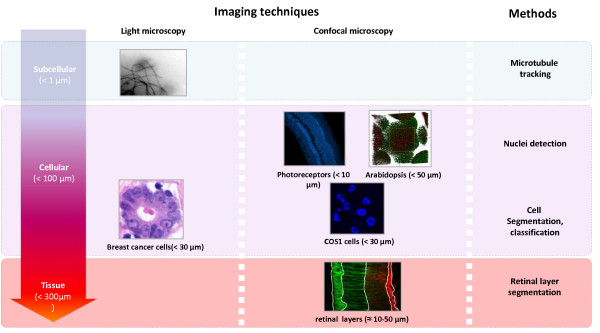
**Dataset**. Example datasets provided in the benchmark are of different scales and modalities.

In the following, we describe both our benchmark infrastructure and comprehensive benchmark datasets for evaluating microscopy image analysis methods. The benchmark includes images with well defined *ground truth*. We provide representative datasets of microbiological structures whose scales range from the subcellular level (*nm*) to the tissue level (*μm*). The collections are obtained through collaborations with domain scientists in molecular, cellular, and developmental biology and in medicine. The datasets highlight some of the current challenges at these varying spatial scales for image analysis (Figure 1). For each of the datasets, we refer the reader to associated analysis methods that are currently integrated or available at our website [18]. We also suggest performance metrics that are used to evaluate the analysis. Finally, we also describe our online benchmark infrastructure [19] which can be used to download the data, upload and analyze the performance of different methods, and software that can be downloaded by researchers for self-evaluation of the analysis methods.

## Results and Discussion

Our biosegmentation benchmark [19] consists of:

• *image datasets *at different scales;

• *ground truth*, manually verified results (e.g. segmentation, cell counting, tracking data);

• *analysis methods*, mostly segmentation methods, cell counting and tracking algorithms;

• *evaluation methods*, image analysis performance measurement;

• *web-accessible infrastructure*.

Identifiable objects in the *image datasets *range from nanometers to microns: images at subcellular, cellular and tissue level are available (see Tables 1, 2 and 3). At the *subcellular level*, we focus on microtubules. At the *cellular level*, we provide a wide range of data. At the *tissue level*, many retinal images have been collected. *Ground truth *is extracted from part of the datasets and is manually verified by domain *experts*. To guarantee a fair comparison of algorithm performance, we split the ground truth into training and testing datasets.

**Table 1:** Subcellular level. Datasets and *ground truth *in the benchmark at subcellular level.

**Type**	**Microtubule tracking**
# images	9 stacks

size (pixel)	512 × 600 × 30

format	.tiff .stk

channels	Rhodamine

condition	Taxol/Docetaxel treated

species	hamster, human (HUVEC)

*ground truth*	1374 traces of microtubules

**Table 2:** Cellular level. Datasets and *ground truth *in the benchmark at cellular level.

**Type**	**Cat retinal photoreceptors**	***Arabidopsis *and cat retinal 3D cells**	**Breast cancer cells**	**COS1 kidney cells**
# images	29 images	10 stacks	58 images	190 images

size (pixel)	512 × 512 (also 768 × 512)	512 × 521 × 50 up to 1056 × 1056 × 30	896 × 768 (also 768 × 512)	1024 × 1024

format	.tiff	.tiff (Flouview),.lsm	.jpg	.tiff

channels	TOPRO	nuclear and membrane stains	H & E stain	Calcein AM (green-alive) Propidium iodide (red-dead) Hoechst (nuclear stain-blue)

condition	normal	normal	malignant/benignant	2, 6, 12,

species	cat	plant, cat	human	monkey

*ground truth*	cell count and centroid	cell centroid	58 binary masks	5 binary masks

**Table 3:** Tissue level. Datasets and *ground truth*in the benchmark at tissue level.

**Type**	**Retina layer detection**
# images	343 images

size (pixel)	300 × 200

format	.bmp .tiff

channels	Rod photoreceptors (*α*Rod opsin) Muller cells (*α*GFAP) Microglia (isolectin B4)

condition	normal, 1-day, 3-day 7-day, 28-day detached

species	cat

*ground truth*	91 layer masks 108 boundary masks

Example implementations of image analysis tools are included for comparing newly developed algorithms. These include detection and tracking at subcellular level; cell counting and segmentation to quantify cellular structures at cellular level and layer segmentation at the tissue level. Researchers can compare the performance of their algorithms through established evaluation metrics such as precision and recall measures. Furthermore, scientists can use this benchmark as a resource for finding the best analysis methods available. All these data and tools are available through a web accessible infrastructure [19].

### Subcellular level

At the subcellular level, the structures within a cell have a typical size of less than 1 *μm*. Cells consist of organelles that are adapted and/or specialized for carrying out one or more vital functions and large assemblies of macromolecules that carry out particular and specialized functions. Such cell structures, which are not formally organelles, include microtubules. Our example dataset at subcellular level consists of time sequence images of microtubules under different experimental conditions. The image analysis challenges at this scale and with the fluorescence imaging acquisition method are typical for *in-vivo *subcellular imaging in that the analysis methods need to cope with high clutter and low signal to noise ratio.

#### Microtubule dataset

**Figure 2:**
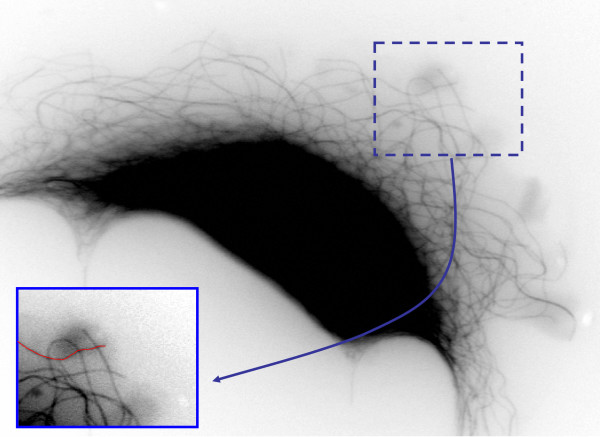
**Microtubule image**. Microtubule image acquired through light microscopy, an enlarged region shows manually tracked microtubule body.

Microtubules are a core component of the cellular cytoskeleton and can function as conveyer belts inside the cells. Researchers believe microtubules play an important role in the study of Alzheimer's and in certain cancers [20]. They move vesicles, granules, organelles like mitochondria, and chromosomes via special attachment proteins. Structurally they are linear polymers of tubulin which is a globular protein. Microtubule growth and shortening, otherwise known as microtubule dynamics, are studied extensively by biologists [21]. Understanding the dynamics of the microtubules under different experimental conditions is important in the study of the above mentioned conditions. Traditionally, microtubule dynamics are obtained by manual microtubule tracking [20,22] (as shown in red in Figure 2). The tracking of the microtubule free ends (tips) allows biologists to compute the growth and shortening statistics which in turn are related to the presence of key proteins such as Tau and its interaction with various drugs.

The microtubule dataset (Table 1) is obtained by transmitted light microscopy at the Feinstein/Wilson Laboratory at University of California, Santa Barbara (UCSB). The microtubule dataset includes 1374 traces which consists of *ground truth *for both microtubule tip location and microtubule bodies.

#### Microtubule tracking

The manual measurements of these microtubules are very labor intensive and time consuming. To obtain an automatic quantitative description of behavior under different experimental conditions, tracing algorithms have been implemented. Due to the limitations in biological sample preparation and inconsistent staining, typical images in live cell studies are noisy and cluttered, making automatic microtubule tracing difficult. Our benchmark implementation includes an automatic method that employs arc-emission Hidden Markov Model for extracting curvilinear structures from live cell fluorescence images [23].

#### Evaluation

**Figure 3:**
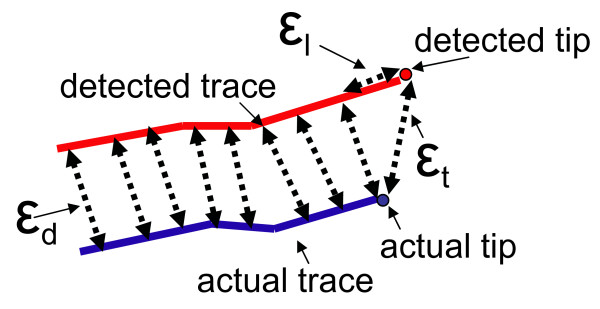
**Microtubule tracing evaluation**. Microtubule tracing evaluation. The blue traces are automatically obtained and the red lines are *ground truth *traces. Tip distance error ϵ_*t*_, trace body distance ϵ_*d *_and length error ϵ_*l*_.

We propose the following three measurements to evaluate microtubule tracing (see Figure 3):

• microtubule tip distance, ϵ_*t*_: tip distance error is the Euclidean distance between the *ground truth *tip to the analysis trace tip,

• microtubule trace body distance, ϵ_*d*_: trace distance error is the average distance from all the points on the *ground truth *to all the points on the trace,

• microtubule length errors, ϵ_*l*_: length difference is simply the difference between the length of the *ground truth *and the trace.

Tracking failure occurs when the above errors are larger than corresponding thresholds:

(1)

(2)

(3)

where the thresholds *τ*_*t*_, *τ*_*b *_and *τ*_*l*_, are empirically set by biologists.

**Figure 4:**
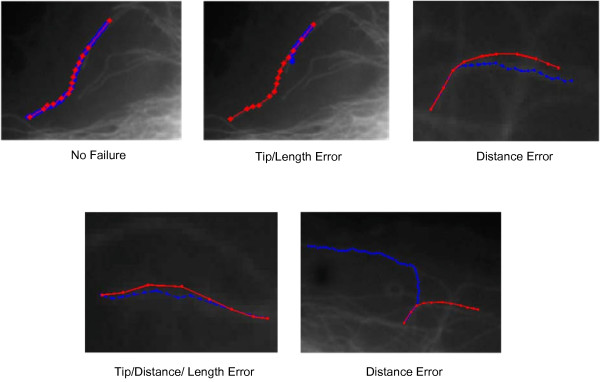
**Microtubule tracing examples**. Microtubule tracing examples. The blue traces are automatically obtained by the algorithm proposed in [23] and the red lines are *ground truth *traces.

For our tracing algorithm described in [23] the failures rate is on average less than 9%. Examples of failure and successful tracking are shown in Figure 4.

### Cellular level

The cell is the structural and functional unit of all known living organisms. A typical cell size is 10 *μm*. Image processing challenges at the cellular level include large variations in cell phenotype, staining intensity variation within and across the images, and occlusions. Cells can grow, reproduce, move and die during many processes, such as wound healing, the immune response and cancer metastasis. One of the common tasks is to count the number of cells or nuclei, particularly in histological sections, and characterize various cell attributes, such as cell density, morphology, size and smoothness of the boundary. In our example datasets, we use cell counting as a feature for estimating cell density in 2D retinal and 3D *Arabidopsis *images, and cell segmentation for studying cell morphology in breast cancer and kidney histopathological images.

#### Photoreceptors in Retinal Images

**Figure 5:**
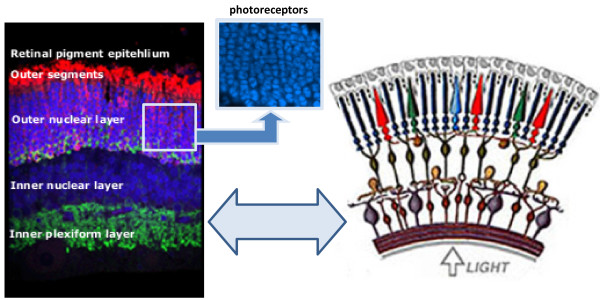
**Retinal layers**. Retinal layers. Confocal microscopy of a vertical section through a cat retina. Each layer has a different structure which consists of the group of cell bodies or synaptic terminals. The photoreceptor cell bodies comprise the ONL. Center top image shows the ONL at higher magnification (boxed area in center image).

The vertebrate retina is a light sensitive part inside the inner layer of the eye. The photoreceptor cells, rods and cones, receive light and transform it into image-forming signals which are transmitted through the optic nerve to the brain. The vertebrate retina is a highly structured tissue and consists of distinct layers as depicted in the cross section Figure 5. The decreasing number of photoreceptor nuclei in the outer nuclear layer (ONL) is one of the characteristics of retina degeneration. The number of photoreceptors in the ONL can decrease due to injury or disease and this may eve result in blindness. The number of photoreceptors in a given section is often used as a measure of effectiveness of a treatment following retinal detachment [24]. Images are typically collected using a laser scanning confocal microscope from tissue sections.

**Figure 6:**
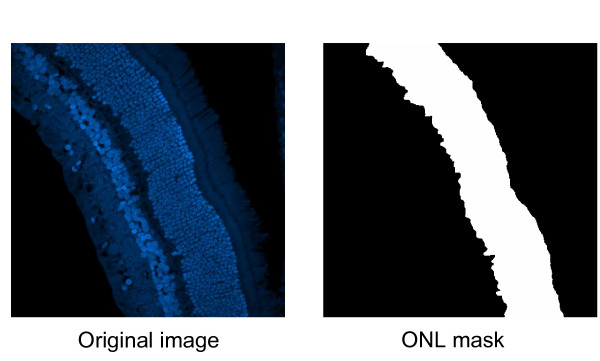
**Example of cell counting in 2D retinal images**. Example of cell counting in 2D retinal images. On the left the original image and on the right the white region denotes the outer nuclear layer (ONL) where the photoreceptor nuclei are located.

The retinal dataset (see Table 2 for details) is collected at the Fisher's Laboratory at UCSB to study retinal degeneration and our benchmark data consists of 29 laser scanning confocal images of normal and 3-day detached feline retinas (9 normal and 20 3-day detached). For each image, the *ground truth *consists of an ONL binary mask and the corresponding manual cell count in the ONL layer provided by three different experts for the same image as depicted in Figure 6.

#### 2D cell nuclei detection

As mentioned above, of particular interest to this collection is detection of cell nuclei. We have implemented in the benchmark system a 2D nuclei detector based on a Laplacian of Gaussian blob detector, see [25] for more details on the method itself.

#### Evaluation

Common ways of evaluating cell/nuclei counting take into account the mismatched counts between detected and *ground truth *nuclei, and/or the displacement of detected nuclei. In 2D analysis evaluation only the counts are available in the *ground truth*.

A simple object count evaluation method was proposed in [25]. The error *E *in the cell count is measured as the ratio between manual counts (obtained by several experts, considered as *ground truth*) and the result of automated detector:

(4)

where *N *is the number of images in the dataset, *ND*_*i *_and  are the number of nuclei automatically detected and the average of manual counting, respectively.

Our nuclei detector [25] applied to the 2D retina dataset gives an error of 3.52% for the nuclei count within the ONL retina layer.

#### Cell Nuclei in 3D Plant Images (*Arabidopsis*)

In plants, meristems are regions of cells capable of division and growth. The live 3D imaging of the *Arabidopsis *meristem has been recently applied in order to analyze the cell lineage and the cell fate during active growth of the shoot meristem [26]. This technique helps to understand the genetic control of the meristem size. Again, cell counts are often used to quantify this process. However, this is an extremely time consuming and laborious task given that a 3D stack consists of approximately 1700 cells.

**Figure 7:**
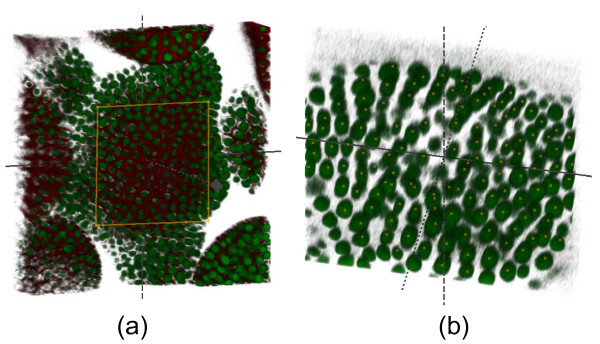
**3D plant images**. 3D laser scanning confocal microscope image of the *Arabidopsis *shoot meristem and its 3D visualization (a). Centroids of detected nuclei of *Arabidopsis *observed in the selected region (b).

Currently we have 10 stacks of images and one annotated *Arabidopsis *laser scanning confocal microscope image generously provided by Meyerwitz's Laboratory at Caltech. The annotated image contains 2 channels with nuclear and membrane stains and is shown in Figure 7(a). The *ground truth *consists of manually detected nuclei centroids as shown in Figure 7(b).

#### 3D cell nuclei detection

The 3D nuclei detection [27] extends our earlier work on 2D detection based on a Laplacian of Gaussian blob detector and it is also integrated into our benchmark. Figure 7(b) shows *Arabidopsis*'s nuclei automatically detected in the selected region of the image shown in Figure 7(a).

#### Evaluation

In 3D, the *ground truth *contains both counts and positions of the nuclei centroids. Thus, we include in this evaluation metric also the nuclei positions available in the 3D *ground truth*. We take into consideration the distance of the detected nuclei from the *ground truth*. The distance error, *E*_*d*_, normalizes the detection error and gives the overall error (*G*_3*D*_) as:

(5)

where false positives (*f*_*p*_) are objects detected in the test image but not present in the ground truth, false negatives (*f*_*n*_) are objects that were not detected in the test image but are present in the *ground truth*. *GT *and *ND *denote, respectively, the *ground truth *(human computed) and automatically detected nuclei coumt. The mean distance () is the mean of all the distances between the detected nuclei locations and their corresponding *ground truth *locations, (*σ*_*d*_) is the standard deviation of these distances, and *d*_*max *_is the max of all the distances. Note that *G*_3*D *_is normalized to [0, 1] and 1 represents the worst case. To quantify the performance of our automatic 3D nuclei detection algorithm [27], we compared its output with *ground truth *manually annotated by two experts and obtained a *G*_3*D *_of 0.1605. When we compared one expert *ground truth *to the other one we obtained a *G*_3*D *_of 0.1260.

#### Breast Cancer Cells

The utility of determining nuclear features for correct cancer diagnosis has been well established in medical studies. Scientists extract a variety of features from nuclei in histopathology imagery of cancer specimens, including size, shape, radiometric properties, texture, and chromatin-specific features. Histopathological images are stained since most cells are essentially transparent, with little or no intrinsic pigment. Certain special stains, which bind selectively to particular components, are used to identify biological structures such as cells. Routine histology uses the stain combination of hematoxylin and eosin, commonly referred to as H&E. In those images, the first step is manual cell segmentation for subsequent classification into benign and malignant cells.

In our benchmark dataset there are 58 H&E stained histopathology images used in breast cancer cell detection from David Rimm's Laboratory at Yale. The *ground truth *is obtained for 50 images including both benign and malignant cells and is described in Table 2.

#### Breast cancer cell segmentation

**Figure 8:**
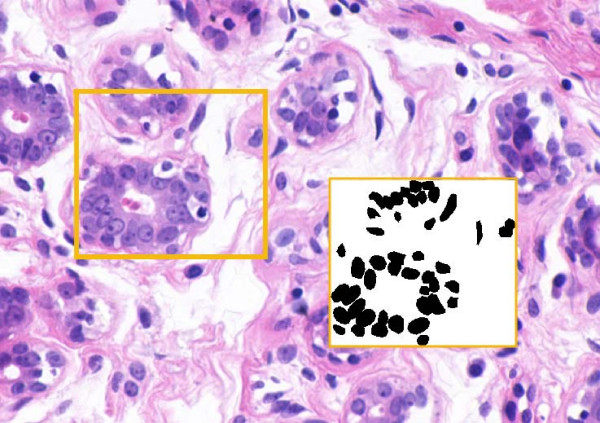
**Breast cancer cell segmentation**. Breast cancer cell segmentation. The original image and its binary mask: a segmented portion of benign cells zoomed on the right.

In [28] a system has been developed which extracts nuclei in histopathology imagery of breast cancer specimens (see Figure 8) based on watershed using as initial condition the results of the 2D cell nuclei detection method mentioned earlier [25].

#### Evaluation

The following metric is defined for the segmentation evaluation of cell nuclei [29]. For the cell segmentation, the size of regions missed, of extraneous regions and their shape are penalized, assuming roughly elliptical shapes for the cells. The metric is given by:

(6)

where *N *is the number of *ground truth *nuclei; *N*_*D *_is the number of nuclei detected by the segmentation algorithm; the weight *α*_1 _can be thought of as the penalty for an over-segmented nucleus; *SR *is the number of segmented regions overlapping the *ground truth *nucleus; *δ*_*SR *_= 1 is the upper limit for number of segmented regions; *PM *is the number of pixels missing; *GT *is the number of pixels in the *ground truth*; *QS*_*PM *_is the "quadrant sum" of the pixels missed; *α*_2 _can be thought of as the penalty regions of pixels missed, penalizing both size and shape; *EP *the number of excess pixel; *α*_3 _is thus the penalty for size and shape of excess pixel regions, and is related to the degree of under-segmentation of the nucleus; *QS*_*EP *_is "quadrant sum" of the excess pixels; the term with *α*_4 _= 1 is simply the detection rate; *ER *as the number of excess segmented regions and *δ*_*ER *_is the fraction of total *ground truth *nuclei that we will allow as excess regions; *α*_5 _is the penalty for excess segmented regions. The metric takes value *P *⊂ [0, 1] and 1 represents the worst segmentation scenario. For a detailed explanation of the metric the reader is referred to [28,29]. Our cell segmenter using the seeds from the 2D cell nuclei segmentation gets a score of *P *= 0.25.

#### Additional dataset

In addition to the above dataset we also have images of kidney cells and *ground truth *corresponding to kidney cell segmentation, but we do not have any associated analysis or evaluation methods for this data. This data was collected by Feinsten's Lab at UCSB to study Alzheimer's disease. Usually manual segmentation provides a reliable alive/dead cell ratio which will test the hypothesis that tau (which is a protein) confers an acute hypersensitivity of microtubules to soluble, oligomeric amyloid-beta and that Taxol, a microtubule-stabilizing drug, provides neuroprotective effects. Because tau is not endogenously expressed, tau effects are easier to study in kidney cancer cells. Kidney cells can easily be transfected with tau. To quantify this phenomenon, COS1 cells (immortalized African monkey kidney cells) are collected through confocal microscopy imaging at the Feinsten's Lab at UCSB.

In the dataset (see Table 2) the images are of both wild-type COS1 cells (non-transfected) and tau transfected COS1 cells and these cells are imaged at 7 different time points after treatment (2 hrs, 6 hrs, 12 hrs, 48 hrs, 72, hrs, and 120 hrs). *Ground truth *has also been collected for 5 images of this dataset and is represented by binary masks.

### Tissue level

**Figure 9:**
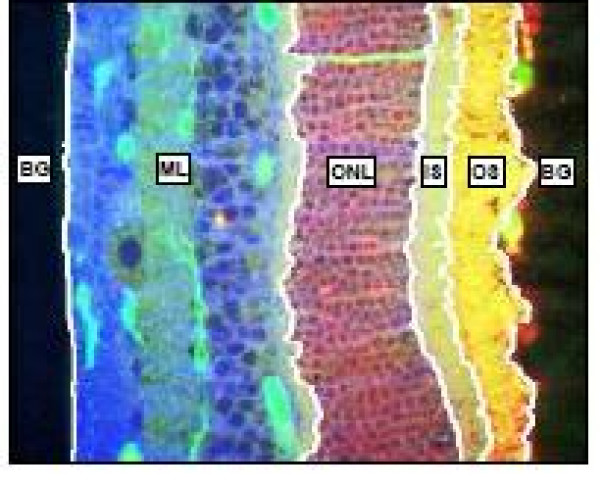
**Retinal layer segmentation example**. Retinal layer segmentation example: the multilayer (ML), the outer nuclear layer (ONL), the inner segments (IS) and the outer segments (OS).

Our tissue level benchmark dataset comes from retinal cross-sections. As discussed in the previous section confocal microscope images of retinas taken during detachment experiments are critical components for understanding the structural and cellular changes of a retina in response to disease or injury. During retinal detachment, layers undergo several changes in morphology. Thus, it is crucial to have a reliable map of the retinal layers. Four major layers of the retina are usually segmented manually to quantify the number of cells in them: the ganglion cell layer (GCL), the inner nuclear layer (INL), the outer nuclear layer (ONL), and the outer segments (OS), as depicted in Figure 9. Hundreds of retinal images [24] and layer *ground truth *are part of the benchmark, see Table 3. Retinal layer segmentation is a challenging problem due to the heterogeneity in retinal images stained with different antibodies.

#### Retinal layer segmentation

**Figure 10:**
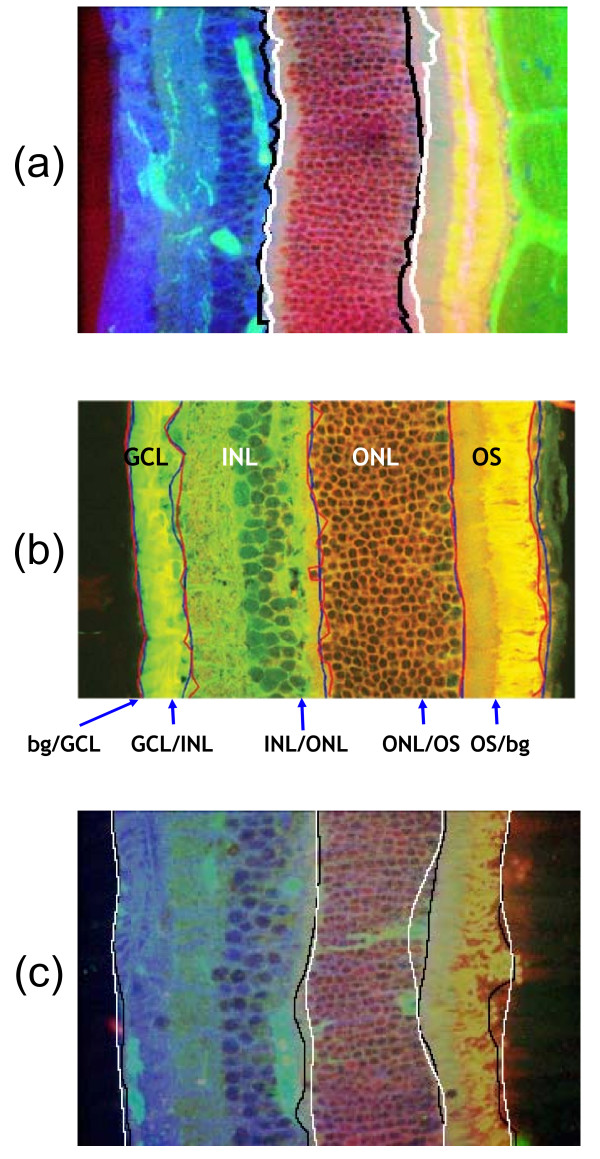
**Examples of layer segmentation**. Examples of layer segmentation results compared to ground truth for feline retinal images normal condition. (a) ONL segmentation: white boundaries detected by [30]'s method compared to black one (*ground truth*); (b) layer segmentation: blue boundaries detected by [31]'s method compared to red one (*ground truth*); (c) INL + GCL, ONL, OS layers segmented by [32] 's method compared to black one (ground truth).

Three automatic retinal layer segmentation methods are integrated into the benchmark. One is a variational segmentation approach based on pairwise pixel similarities [30]. This method exploits prior information (reference image) and a dissimilarity measure between the pixels of the reference image and the pixels of the image to be segmented. The second segmentation algorithm uses parametric active contour to detect the boundaries between layers [31]. The third method [32] is a segmentation based on non rigid registration using thin plate splines, which assumes a non rigid transformation of the layer boundaries of a training retinal image in order to segment the test image. An example of the segmentation results and *ground truth *are respectively shown in Figures 10(a), (b) and 10(c).

#### Evaluation

For boundary evaluation, the distance between the *ground truth *boundary pixels and computed boundaries for each layer is computed. For layer evaluation, several measures are implemented: precision (*p*) is the ratio between true positive and automatically detected pixels; recall or sensitivity (*r*) is the ratio between true positive pixels and *ground truth*; F-measure (*F*) is the harmonic mean between precision and recall for each layer defined as:

(7)

the weighted F-measure (*F*_*w*_) is the sum of the F-measure scores for each layer *i *in proportion to their area *A*_*i *_of the total area *A*_*tot*_:

(8)

This modified F measure allows for weighting more segmentation errors in larger layers.

Our best performing method [30] gives a F-measure around 88% when applied on the dataset. The average distance between boundary pixels in the computed and *ground truth *data using the boundary detection method from [31], averaged over all experimental conditions, is 9.52 pixels.

## Conclusion

Benchmark datasets often have a strong positive effect in advancing the state of the art of image analysis algorithms.

The benefits of benchmarking include a stronger consensus on the research goals, collaboration between laboratories, transparency of research results, and rapid technical progress. Our benchmark provides unique, publicly available datasets as well as image analysis tools and evaluation methods. The benchmark infrastructure avoids the burden of choosing datasets for testing algorithms, reimplementing analysis methods and evaluation metrics for comparison.

We hope that our benchmark will help researchers to validate, test and improve their algorithms, as well as provide biologists a guidance of algorithms' limitations and capabilities. The benchmark datasets and methods are available online [19]. Analysis results can be uploaded directly and automatically evaluated. The benchmark data that we describe is by no means complete, given the complexity and diversity of the bio-samples and imaging modalities. By making this infrastructure easily accessible to the community, we hope that the collections and analysis methids will grow over time. Users are encouraged to submit datasets, associated *ground truth*, and analysis results for evaluation. Moreover, user contributed analysis (*e.g. *segmentation) algorithms and evaluation methods will be integrated upon request.

## Availability and Requirements

It is generally acknowledged that making fair comparisons between different methods is quite difficult, particularly in the absence of well established datasets. In addition, even if such datasets are available, often the researchers are left to implement other methods on their own in order to make such comparisons. Newly developed algorithms are often tested against relatively limited datasets. Keeping these limitations in mind, we have been developing the UCSB Bisque infrastructure [18,33] whose primary goal is to facilitate a tighter integration of datasets and analysis methods.

In the following, we outline the Bisque [33] components that are relevant to the benchmarking effort. All of the working modules and datasets are available from our bioimage informatics website [19]. From the website users can download the different datasets discussed in this paper and associated *ground truth *data. Each dataset includes a complete set of original images to process, a document in *XML *format and an example of *ground truth*. The *xml *structure follows Bisque standards [18] and examples for metadata and graphical annotations are presented in the Appendix. A complete description and formatting of metadata for each dataset is given in [19].

Two evaluation options are available to the users (the evaluation procedures implement the scoring methods discussed in the previous sections for the different datasets):

• Matlab code for evaluation is available for download. Users can download this code and self evaluate the performance of the analysis methods. The evaluation requires that the results are stored in a certain format and a detailed description of the file formats are also provided on Bisque,

• users can also evaluate the performance using our web-based evaluation module. In order to use the web-based evaluation the users must first register into Bisque. They need to first upload their results, one image at the time in the correct format to the web-based evaluator and the evaluation results will be automatically displayed on the web site. This option will also allow the result to be stored on our benchmark website and made available to the registered users.

## Abbreviations

**Caltech**: California Institute of Technology; **COS**: CV-1 (simian) in Origin, and carrying the SV40 genetic material; **CT**: Computed Tomography; **FRGC**: Face Recognition Grand Challenge; **FRVT**: Face Recognition Vendor Test; **GCL**: Ganglion Cell Layer; **H&E**: Hematoxylin Eosin; **INL**: Inner Nuclear Layer; **IS**: Inner Segments; **MRI**: Magnetic Resonance Imaging; **ML**: MultiLayer; **ONL**: Outer Nuclear Layer; **OPL**: Outer Photoreceptor Layer; **OS**: Outer Segments; **UCSB**: University of California Santa Barbara.

## Authors' contributions

EDG developed the initial benchmark, web site and tested the image analysis code. She also developed the different evaluation measures. BO developed the Matlab toolbox to access the Bisque database system. BO, DF and KK conceived and developed the flexible database infrastructure and access tools used by the benchmark. BM conceived the project and coordinated the research and development activities. BM and KK refined the the manuscript drafted by EDG, OB and DF. All the authors read and approved the manuscript.

## Appendix

*Flexible data model*: The benchmark uses a flexible metadata model based on tag documents to store and process *ground truth *and resultant data. A tag is a named field with an associated value. The tags themselves may be nested, and include values that are strings, numbers, other documents or list. For example, the 3D cell counting document has:

<benchmark type="3D nuclei"> <image name="1. tiff " src ="1. tiff ">

   <gobject name="my_algorithm " type="NucleiDetector3D_automatic">

      <gobject name="1" type="point">

         <vertex x="0" y="1020" z="0"/>

      </gobject >

      <gobject name="2" type="point">

         <vertex x="1048" y="941" z="4"/>

      </gobject >

      <gobject name="3" type="point">

         <vertex x="871" y="1046" z="2"/>

      </gobject >

      ...

   </gobject >

</image> </benchmark>

The benchmark uses also an extensible metadata format for graphical annotations that has a number of graphical primitives and can be extended by new object types and object properties. Graphical annotations are termed *gobject *s and are used, to represent *ground truth *objects in the dataset. The following is an example *gobject *description for microtubules:

<gobject name="gt " type="mt_gt" >

<tag name="expert " value="expert_name"/>

<tag name="tube_id " value ="1"/>

   <polyline type="polyline " name="polyline">

      <vertex x="235.503009" y="170.699054" index="0" t ="0.000000"/>

      <vertex x="246.143594" y="174.614789" index="1" t ="0.000000"/>

</polyline >

<polyline type="polylin e " name="polyline">

      <vertex x="235.503009" y="170.699054" index="0" t ="1.000000"/>

      <vertex x="250.144454" y="175.891660" index="1" t ="1.000000"/>

</polyline >

...
